# Serum GAA as a Possible Biomarker of Exhaustive Exercise?

**DOI:** 10.3389/fphys.2019.01506

**Published:** 2019-12-12

**Authors:** Sergej M. Ostojic, Valdemar Stajer, Laszlo Ratgeber, Jozsef Betlehem, Pongras Acs, Andras Olah

**Affiliations:** ^1^Faculty of Sport and Physical Education, University of Novi Sad, Novi Sad, Serbia; ^2^Faculty of Health Sciences, University of Pecs, Pécs, Hungary

**Keywords:** creatine, bioenergetics, GAA, exhaustion, resistance exercise, recovery

## Introduction

Exercise alters human homeostasis by different means, with energy metabolism particularly affected by heavy and strenuous activities. From a substantial increase in an expenditure of cellular energy to an up-regulation of proteins involved in all aspects of energy turnover (Smiles et al., 2016), exhaustive exercise is consistently accompanied by considerable changes in various indicators of energy metabolism in the circulation and energy-demanding tissues, such as the brain, heart, or skeletal muscle (Kastellorizios and Burgess, 2015). Specifically, a plethora of blood-based biomarkers have emerged in recent years as effective screening tools used to monitor exhaustive exercise-induced changes in bioenergetics (Lee et al., 2017). At the same time, it remains puzzling why the indices of creatine metabolism in the blood are rarely used in routine screening during exercise, having in mind a fact that creatine utilization plays a major role during activities in which short bursts of intense energy are required (Wallimann et al., 2011). Besides other creatine-related compounds, guanidinoacetic acid (GAA), a direct endogenous precursor of creatine, might be particularly sensible to exhaustive exercise. This opinion paper summarizes the latest findings for advancing serum GAA as a possible innovative biomarker of exhaustive exercise, as well as explores the shortcomings and challenges that need to be addressed before the translation of its use in routine practice. We selected all human studies know to the authors that demonstrated notable changes in serum GAA after either aerobic or resistance exercise to exhaustion.

## Serum GAA and Exhaustive Exercise

**Figure 1:**
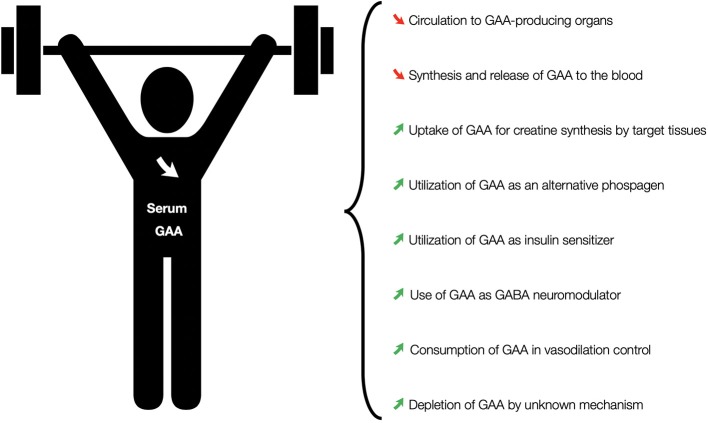
Possible mechanisms involved in exercise-induced changes in serum GAA levels.

Sotgia et al. (2007) were among the first who demonstrated a change in serum GAA to heavy exercise. Why the main aim of this pioneering study was to evaluate whether the modification in blood homocysteine after exercise is explainable in the light of the common connection of physical activity and homocysteine to creatine, the authors also reported that sedentary volunteers and athletes experienced a significant drop in serum GAA (16.1% on average) after a session of an incremental cycle ergometer test till exhaustion. Our group confirmed the effects of a single brief bout of strenuous exercise on circulating concentrations of GAA in healthy volunteers subjected to running to exhaustion and free-weight bench-press to volitional failure (Stajer et al., 2016), with running induced a decrease in serum GAA by 20.1% while resistance exercise reduced GAA levels by 11.7%. Either a transient or discernible decline in circulating GAA was also demonstrated after strenuous upper body exercise (Al Fazazi et al., 2019), and following running at anaerobic threshold until exhaustion (Stajer et al., 2019). GAA response appears to be depended on the mode of exhaustive exercise (aerobic vs. resistance), the type of aerobic activity (running vs. cycling), a model of load intensity over time (continuous vs. intermittent), and the duration of an individual exercise session. According to previous human studies, serum GAA levels are largely affected by a single short episode (~6 min) of continuous RAMP aerobic running until exhaustion, while GAA concentrations remain less responsive to other exercise modalities. This heavy exercise-induced reduction in circulating GAA might be due to several possible mechanisms (Figure 1). Due to the fact that endogenous GAA is mainly produced by the kidney, liver, and pancreas (da Silva et al., 2009), an exercise-driven diminution of systemic circulation to these organs might negatively affect GAA production and/or discharge, translating to lower GAA levels. Second, GAA from the blood could be taken up during exercise to an increasing extent by active cells (such as skeletal or cardiac myocytes), either as a metabolic compound that yields creatine (Wyss and Kaddurah-Daouk, 2000) or as a possible substrate for creatine kinase and an alternative phosphagen in high energy-demanding conditions (Kan et al., 2004). On top of that, circulating GAA could be subsumed as an insulin sensitizer, GABA neuromodulator or a vasodilation agent (Ostojic, 2015), and/or consumed by an unknown metabolic pathway instigated by heavy exercise. For example, high-intensity exercise appears to increase insulin sensitivity and secretion (Søgaard et al., 2018), with extra insulin may perturb glomerular filtration and affect GAA excretion (ter Maaten et al., 1999). To address this, a tracer study with isotopically labeled GAA (such as guanidino-[^13^C_2_]acetic acid) is highly warranted to track down a feasible tissue-specific performance of this compound for the duration of exhaustive exercise. Theoretically, alterations in circulating GAA might help us to better understand a complex interplay between changes in circulation and bioenergetics among different organs during heavy exercise.

Once dropped after an exercise session, GAA levels seem to steadily return to pre-exercise values. Al Fazazi et al. (2019) recently monitored 24-h dynamics for serum biomarkers of creatine metabolism after an acute session of exhaustive resistance exercise. Serum GAA dropped for 11.1% immediately after an exercise session, with concentrations remained significantly reduced at both 15- and 60-min post-exercise follow-ups for 9.9 and 5.1%, respectively. However, GAA concentrations sampled at 24-h post-exercise period restored to the baseline values, suggesting a recovered GAA homeostasis for the next exhaustive exercise session set at a following day (Al Fazazi et al., 2019). This delicate changes in circulating GAA could be perhaps more convenient for a time-sensitive evaluation of post-exhaustive exercise recovery, as compared to traditional biomarkers employed for recovery surveillance (Lee et al., 2017).

## Open Questions and Challenges

The vast majority of contemporary trials evaluated exhaustive exercise-driven changes in serum GAA in active healthy men, while no up-to-date information has been available for other relevant groups (e.g., sedentary populations, clinical patients, women, different age groups). In particular, having in mind that GAA-synthesizing enzyme (L-arginine:glycine amidinotransferase) is stimulated by testosterone while the main GAA transporter (SLC6AB) is down-regulated by estrogen (Joncquel-Chevalier Curt et al., 2013) it seems plausible to examine a possible gender-related difference in serum GAA response to heavy exercise. A recent study also demonstrated comparatively heterogenous individual responses in circulating GAA levels during the course of the treadmill run (Stajer et al., 2019), putting forward a possible responder-non-responder feedback for GAA turnover during exhaustive exercise, which warrants further investigation before advancing it for a general population. A comparison between serum GAA and traditional biochemical markers of exhaustive exercise revealed equivocal results. Exercise-induced changes in GAA levels appear to poorly correlate with blood lactate, muscle-specific creatine kinase, lactate dehydrogenase, and interleukin 6 evaluated for the duration of exhaustive exercise (Al Fazazi et al., 2019; Stajer et al., 2019). This might be a result of separate domains of exercise physiology evaluated (e.g., bioenergetics vs. muscular damage and inflammation), and/or time-sensitive variation in specific responses to exhaustive exercise, with serum GAA perhaps immediately respond to exhaustive exercise while other biomarkers are characterized by a relatively slow release from the target cells. On the other hand, a moderate-to-strong positive correlation was demonstrated between changes in serum cortisol and GAA levels during exercise (*r* = 0.79) (Stajer et al., 2019), implying a link between stress hormones and impaired bioenergetics. However, does cortisol have an effect on the transport of GAA from the blood to the skeletal muscle, acting as a modulator of amino acid uptake by high-energy output tissues (Christiansen et al., 2007), or GAA as a creatine precursor adjusts cortisol response during strenuous exercise (Dobgenski et al., 2014) remain unknown at the moment. Further studies are needed to evaluate possible impact of circulating cortisol on SLC6A8 expression and activity, along with potential impact of GAA on cortisol synthesis in the adrenal cortex (Stajer et al., 2019).

## Conclusion

GAA is an important intermediate in cellular bioenergetics, with a synthesis of the main energy “buffer” (creatine) being its only metabolic fate. It appears that GAA availability in the circulation could be modulated by physical exercise, perhaps due to an exercise-induced disbalance in tissue-specific GAA release and removal. Initial studies suggest that strenuous activities diminish circulating GAA levels in a magnitude-depended manner thus putting forward its use as a prospective biomarker of exhaustive exercise. Nevertheless, the exact mechanism of GAA utilization during heavy exercise needs to be further clarified and a more detailed exposure-response relationship (quantified by exercise duration, frequency, absolute, and relative intensity) is required before recommending serum GAA as an accurate tool for general practice in exercise physiology and medicine. To achieve this, further carefully designed exercise intervention studies in both athletic and clinical settings are necessary to drive knowledge gain in this exciting area.

## Author Contributions

SO drafted the manuscript. VS, LR, JB, PA, and AO performed a critical revision of the manuscript.

### Conflict of Interest

The authors declare that the research was conducted in the absence of any commercial or financial relationships that could be construed as a potential conflict of interest.
